# Building-block synthesis as a determinant of antibiotic persistence

**DOI:** 10.1038/s41467-026-78277-3

**Published:** 2026-09-28

**Authors:** Anaïs Padellec, Charlotte Michaux, Séverin Ronneau

**Affiliations:** INSERM U1230, University of Rennes, Rennes, France

**Keywords:** Bacteriology, Bacterial physiology, Antimicrobial resistance

## Abstract

Reduced antibiotic target activity is a major driver of antibiotic treatment failure. While this has mostly been linked to the presence of low-energy bacteria, a recent study by Taher and colleagues suggests that reduced building-block synthesis can also promote antibiotic survival during nitrogen starvation — a route distinct from energy limitation.

Drug-susceptible bacteria should, in theory, be eliminated by bactericidal antibiotics. However, a subpopulation of cells known as antibiotic persisters can survive during infection and drive relapse after treatment ends^1^. Because most bactericidal antibiotics act on essential cellular processes to induce lethal damage, transient reduction of drug target activity has been proposed as a central driver of antibiotic persistence^2^. This reduced activity has largely been attributed to low cellular energy, arising from stochastic variation in energy-metabolism enzymes, nutrient scarcity, or exposure to reactive oxygen and nitrogen species that ultimately impair bacterial respiration^3–5^. ATP depletion as a driver of antibiotic survival is well established across pathogens—from *Escherichia coli* and *Salmonella enterica* to *Staphylococcus aureus*—in both laboratory and infection settings^6^.

In their new study^7^, Taher and colleagues propose that nitrogen starvation promotes antibiotic survival not by lowering ATP levels, but through a single metabolic node that constrains the precursor pools for DNA replication and peptidoglycan synthesis, on which fluoroquinolones and β-lactams, respectively, depend for their lethality. The authors show that nitrogen-starved bacteria sharply decrease expression of PyrBI, which catalyzes the first step of pyrimidine biosynthesis from carbamoyl phosphate. Using a mother machine and targeted overexpression of individual pathway enzymes, they observe that cell-to-cell variation in PyrBI during nitrogen starvation correlates with survival. Cells with low PyrBI expression are more likely to survive antibiotic treatment, and forcing uniformly high expression collapses this advantage (Fig. 1A). Importantly, overexpression of other nitrogen-related genes, such as *glnA*, encoding glutamine synthetase, or *carAB*, encoding the enzyme providing carbamoyl phosphate to PyrBI, does not resensitize cells to antibiotics. In agreement with the role of PyrBI in antibiotic persistence, pharmacological inhibition of PyrBI is sufficient to increase the fraction of cells surviving meropenem and ciprofloxacin treatment under conditions where nitrogen is available. Finally, Taher and colleagues show that nitrogen starvation shapes biofilm architecture, with high-PyrBI-expressing cells enriched in the biofilm core, decreasing towards the periphery. They suggest that this amplification of cell-to-cell variability in the biofilm environment might contribute to the difficulty of eradicating biofilms in nitrogen-limited environments.

**Fig. 1:**
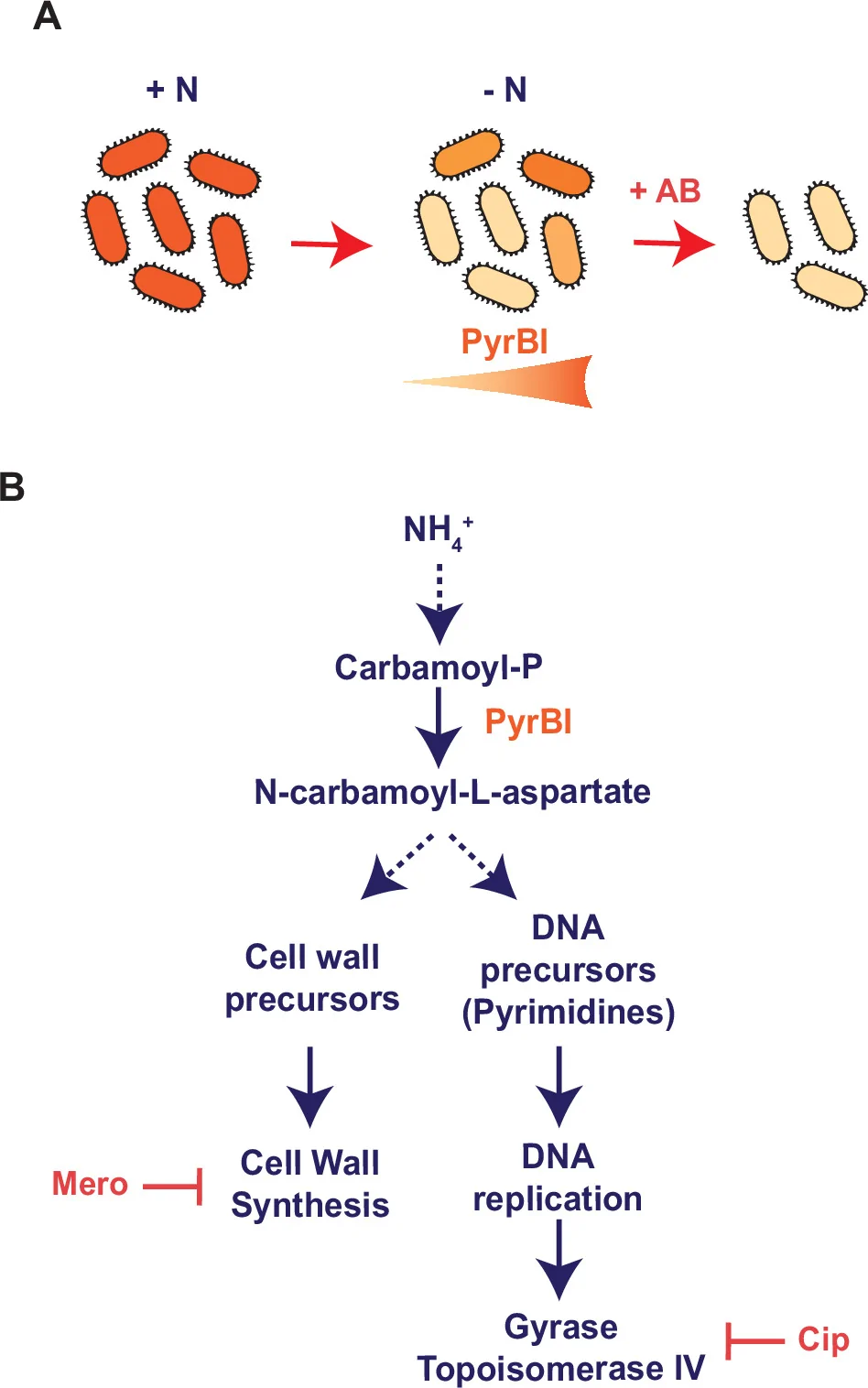
Variation in PyrBI expression during nitrogen starvation contributes to antibiotic persistence. **A** Nitrogen starvation increases cell-to-cell variability in PyrBI expression, increasing the frequency of persister cells within the population; cells with low PyrBI levels are more likely to survive antibiotic treatment (+AB). **B** PyrBI acts as a metabolic node linking nitrogen availability to the synthesis of DNA precursors (pyrimidines) and cell-wall precursors, thereby constraining the activity of the targets of ciprofloxacin (Cip) and meropenem (Mero). Dashed arrows represent indirect metabolic steps involving additional, unrepresented enzymes.

But how might cell-to-cell variation in PyrBI limit the lethality of both β-lactams and fluoroquinolones during nitrogen starvation? From a metabolic perspective, PyrBI ultimately feeds the UTP pool, which branches into two essential outputs: pyrimidine nucleotides required for DNA replication, and UDP-activated sugars that serve as building blocks for peptidoglycan synthesis (Fig. 1B). Although the authors did not directly quantify DNA or cell-wall precursors, a single metabolic bottleneck could in principle constrain both antibiotic-relevant pathways at once, without requiring any change in ATP availability. This provides a unifying explanation, as limitation of a common precursor could underlie tolerance to both drug classes rather than requiring separate mechanisms.

Taken together, the paper offers a compelling new perspective on the relationship between target activity and antibiotic susceptibility. Rather than treating ATP depletion as the exclusive route to a low-activity target, Taher and colleagues suggest an interesting alternative: the substrates required for essential cellular processes may instead be missing in persister cells—a distinct route to the same phenotypic endpoint.

While this study pushes the persister field forward by supporting an alternative route that does not depend directly on energy metabolism, several questions remain open. For instance, the authors conclude that (p)ppGpp does not contribute to antibiotic tolerance during nitrogen starvation, although previous studies point in that direction^8^. They develop a (p)ppGpp reporter and validate it using serine hydroxamate (SHX) to trigger the stringent response—a reasonable first step that would benefit from direct validation by TLC-based (p)ppGpp quantification during nitrogen starvation, a well-established trigger of (p)ppGpp accumulation^8,9^. Whether persistence under these conditions is truly independent of (p)ppGpp, or occurs alongside it, therefore remains an open question—one with particular relevance given the recurring role of (p)ppGpp in starvation-induced persistence, notably in biofilms^10,11^.

Beyond PyrBI itself, the broader transcriptional response to nitrogen starvation is also worth considering, as many additional genes are differentially expressed during nitrogen starvation and are expected to behave like PyrBI in mother machine experiments. Notably, the authors show in their transcriptomic data that *gyrA*, *mrcA* and *mrdA*—the direct targets of fluoroquinolones and β-lactams—are among the genes significantly downregulated under nitrogen starvation. This raises the possibility that precursor limitation and reduced target abundance act together to decrease target activity. Still, reduction of target activity does not tell the whole story of antibiotic survival. Although overexpression of PyrBI alone resensitizes 90% of nitrogen starvation-induced persisters to antibiotics, this experiment does not rule out the contribution of additional factors to persister formation, survival, and regrowth following drug exposure. Consistent with this, >10⁵ persisters per mL still form colonies after 24 h of treatment, which cannot be accounted for by PyrBI variation alone. For instance, bacteria have access to other safeguards that influence survival independently of target activity. Persisters surviving fluoroquinolones exhibit extensive antibiotic-induced damage that they must repair through the SOS response to regrow^12,13^. Similarly, bacteria can reduce antibiotic concentration near their targets through efflux machinery, as shown for both β-lactams and fluoroquinolones^14,15^. These mechanisms, which do not allow bacteria to proliferate in the presence of antibiotics but rather allow them to survive and regrow after treatment, directly contribute to antibiotic persistence and might also be affected by nitrogen starvation.

Importantly, this work paves the way to several future challenges. First, it remains to be established how findings from the *E. coli* laboratory strain used here translate to clinically relevant pathogens, where antibiotic tolerance already poses a major clinical burden. Second, whether nitrogen starvation is actually encountered by pathogens during infection—and to what extent—needs more investigation in vivo, given the heterogeneous and dynamic nutrient landscapes that defined minimal media cannot fully capture. Several niches encountered during infection are characterized by nutrient scarcity, and nitrogen availability specifically remains poorly characterized across these environments compared to oxygen or carbon limitation. Third, a translational question follows naturally from the mechanism: could co-administering antibiotics with the limiting metabolic precursors restore target activity and improve efficacy against persisters? Answering these questions will determine whether this new perspective on target inactivity remains a conceptual advance or becomes a genuine therapeutic entry point.
